# Learning Integrated Health System to Mobilize Context-Adapted Knowledge With a Wiki Platform to Improve the Transitions of Frail Seniors From Hospitals and Emergency Departments to the Community (LEARNING WISDOM): Protocol for a Mixed-Methods Implementation Study

**DOI:** 10.2196/17363

**Published:** 2020-08-05

**Authors:** Patrick Michel Archambault, Josée Rivard, Pascal Y Smith, Samir Sinha, Michèle Morin, Annie LeBlanc, Yves Couturier, Isabelle Pelletier, El Kebir Ghandour, France Légaré, Jean-Louis Denis, Don Melady, Daniel Paré, Josée Chouinard, Chantal Kroon, Maxime Huot-Lavoie, Laetitia Bert, Holly O Witteman, Audrey-Anne Brousseau, Clémence Dallaire, Marie-Josée Sirois, Marcel Émond, Richard Fleet, Sam Chandavong, Network Of Canadian Emergency Researchers

**Affiliations:** 1 Department of Family and Emergency Medicine Université Laval Québec, QC Canada; 2 Centre intégré de santé et de services sociaux de Chaudière-Appalaches Ste-Marie, QC Canada; 3 VITAM - Centre de recherche en santé durable Québec, QC Canada; 4 Department of Anesthesiology and Critical Care Medicine Division of Critical Care Medicine Université Laval Québec, QC Canada; 5 Centre de recherche intégrée pour un système apprenant en santé et services sociaux Centre intégré de santé et de services sociaux de Chaudière-Appalaches Lévis, QC Canada; 6 Department of Medicine Sinai Health System Toronto, ON Canada; 7 Department of Medicine University Health Network Toronto, QC Canada; 8 Department of Medicine University of Toronto Toronto, QC Canada; 9 Department of Medicine Faculty of Medicine Université Laval Québec, QC Canada; 10 Department of Social Work University of Sherbrooke Sherbrooke, QC Canada; 11 Institut national d'excellence en sante et en services sociaux Québec, QC Canada; 12 Centre intégré universitaire de santé et de services sociaux de la Capitale-Nationale (CIUSSS-CN) Québec, QC Canada; 13 Canada Research Chair in Shared Decision Making and Knowledge Translation Université Laval Québec, QC Canada; 14 Département de gestion, d’évaluation et de politique de santé École de santé publique Université de Montréal Montreal, QC Canada; 15 Schwartz-Reisman Emergency Medicine Institute Mount Sinai Hospital Toronto, ON Canada; 16 Department of Family and Community Medicine University of Toronto Toronto, ON Canada; 17 Faculty of Medicine Université Laval Québec, QC Canada; 18 Faculty of Nursing Université Laval Québec, QC Canada; 19 Office of Education and Professional Development Faculty of Medicine Université Laval Québec, QC Canada; 20 CHU de Québec-Université Laval Québec, QC Canada; 21 Centre intégré universitaire de santé et de services sociaux de l'Estrie - CHUS Sherbrooke, QC Canada; 22 Centre d'excellence sur le vieillissement du Québec Hôpital du Saint-Sacrement Québec, QC Canada; 23 Département de réadaptation Faculté de médecine Université Laval Québec, QC Canada; 24 Network of Canadian Emergency Researchers Ottawa, ON Canada

**Keywords:** implementation science, knowledge translation, context adaptation, interrupted time series, care transitions, elderly, older persons, health care utilization, frailty, learning health systems, Wiki, collaborative writing applications

## Abstract

**Background:**

Elderly patients discharged from hospital experience fragmented care, repeated and lengthy emergency department (ED) visits, relapse into their earlier condition, and rapid cognitive and functional decline. The Acute Care for Elders (ACE) program at Mount Sinai Hospital in Toronto, Canada uses innovative strategies, such as transition coaches, to improve the care transition experiences of frail elderly patients. The ACE program reduced the lengths of hospital stay and readmission for elderly patients, increased patient satisfaction, and saved the health care system over Can $4.2 million (US $2.6 million) in 2014. In 2016, a context-adapted ACE program was implemented at one hospital in the Centre intégré de santé et de services sociaux de Chaudière-Appalaches (CISSS-CA) with a focus on improving transitions between hospitals and the community. The quality improvement project used an intervention strategy based on iterative user-centered design prototyping and a “Wiki-suite” (free web-based database containing evidence-based knowledge tools) to engage multiple stakeholders.

**Objective:**

The objectives of this study are to (1) implement a context-adapted CISSS-CA ACE program in four hospitals in the CISSS-CA and measure its impact on patient-, caregiver-, clinical-, and hospital-level outcomes; (2) identify underlying mechanisms by which our context-adapted CISSS-CA ACE program improves care transitions for the elderly; and (3) identify underlying mechanisms by which the Wiki-suite contributes to context-adaptation and local uptake of knowledge tools.

**Methods:**

Objective 1 will involve staggered implementation of the context-adapted CISSS-CA ACE program across the four CISSS-CA sites and interrupted time series to measure the impact on hospital-, patient-, and caregiver-level outcomes. Objectives 2 and 3 will involve a parallel mixed-methods process evaluation study to understand the mechanisms by which our context-adapted CISSS-CA ACE program improves care transitions for the elderly and by which our Wiki-suite contributes to adaptation, implementation, and scaling up of geriatric knowledge tools.

**Results:**

Data collection started in January 2019. As of January 2020, we enrolled 1635 patients and 529 caregivers from the four participating hospitals. Data collection is projected to be completed in January 2022. Data analysis has not yet begun. Results are expected to be published in 2022. Expected results will be presented to different key internal stakeholders to better support the effort and resources deployed in the transition of seniors. Through key interventions focused on seniors, we are expecting to increase patient satisfaction and quality of care and reduce readmission and ED revisit.

**Conclusions:**

This study will provide evidence on effective knowledge translation strategies to adapt best practices to the local context in the transition of care for elderly people. The knowledge generated through this project will support future scale-up of the ACE program and our wiki methodology in other settings in Canada.

**Trial Registration:**

ClinicalTrials.gov NCT04093245; https://clinicaltrials.gov/ct2/show/NCT04093245.

**International Registered Report Identifier (IRRID):**

DERR1-10.2196/17363

## Introduction

### Background

In 2019, more than one in six Canadians were aged 65 years or older. These aging Canadians will account for 20% of the population by 2024 [1]. It is a challenge for our health care system to meet the growing needs of the aging population, whose members often have chronic conditions, take multiple medications, and receive care from multiple providers. Moreover, they are typically frequent health care users, with the system spending more on them than on any other segment of the population [2]. Seniors represent a third of all patients consulting emergency departments (EDs) [3-7]. Seniors are especially vulnerable to health system failures; one-third report experiencing care coordination problems, with the most important problems according to patients being gaps in hospital discharge planning [8,9] and long waiting lists to receive home care [10,11]. Seniors and their caregivers are obliged to manage their own care through a broken care continuum [12-14]. Discharge adverse events result in unplanned readmissions [15,16], which occur after 10%-30% of medical admissions [16-28], and loss of physical, functional, and/or cognitive capacity [29-34]. Added to poor health outcomes are patient and staff distress [35,36] and increasing lawsuits concerning inadequate discharge planning [37].

### Acute Care for Elders Program: Best Practice Guidelines for Elder Care Transitions

Improving care transitions for seniors requires a multifaceted integrated approach based upon best practices [11,38], such as the Acute Care for Elders (ACE) program developed by Mount Sinai Hospital in Toronto [39-41]. Over the last decade, Mount Sinai has become Canada’s most widely recognized elder-friendly hospital, implementing evidence-informed point-of-care interventions to improve patient, provider, and system outcomes for frail older persons [2]. Supported by systematic reviews [42-48] and randomized clinical trials [49-52], the ACE care transition program is based on interprofessional interventions to enhance postdischarge care. Comparing performance in the baseline year 2009 with that in 2014, Mount Sinai reduced the total length of stay (from 12 to 8 days), reduced the alternate level of care days by 20%, reduced readmissions within 30 days (from 15% to 13%), improved the rate of patients returning home as opposed to other institutional settings (from 71% to 79%), and increased the rate of patient satisfaction (from 95% to 97%). These improvements resulted in an estimated Can $4.2 million (US $2.6 million) in savings in 2014 [53].

Unfortunately, the ACE best practice guidelines have only been implemented in a few dozen acute care organizations around the world [54]. A major barrier to their implementation is that these guidelines and tools cannot be easily transferred into different cultural, organizational, and technical contexts. Knowledge producers (researchers) and knowledge users (patients/caregivers, clinicians, and decision makers) lack effective interventions to adapt knowledge to the local context, a crucial step in the knowledge-to-action (KTA) framework [55,56]. Knowledge users lack the skills, resources, or institutional culture necessary to apply knowledge locally and support their institutions to learn new ways of operating. Researchers have been challenged to find new solutions that support the involvement of local knowledge users in adapting knowledge tools to their contexts [57-61]. Although local adaptation is a key step of the KTA framework, little is known about how to accomplish this step effectively [55,56,62,63]. New solutions are needed to support the involvement of knowledge users in adapting knowledge tools to their contexts [56-60].

### Two Novel Knowledge Translation Interventions to Adapt the ACE Program to the Local Context

In 2016, the *Centre intégré de santé et de services sociaux de Chaudière-Appalaches* (CISSS-CA) was selected by the Canadian Foundation for Healthcare Improvement (CFHI), the Canadian Frailty Network, and Mount Sinai Hospital to implement and adapt the ACE program in the local context. This adaptation also had to be performed in synergy with Quebec’s provincial elder-friendly best practices, the *Approche adaptée à la personne âgée en milieu hospitalier* (senior-friendly hospital care) [64].

A research team led by the first author (PA) and by the Chief Executive Officer (CEO) of the CISSS-CA (DP) has been using two novel knowledge translation (KT) interventions (WikiTrauma and Wiki101 [“the Wiki-suite”]) to engage knowledge users in adapting the ACE program in the local context at the Hôtel-Dieu de Lévis, one of the four acute care hospitals within the CISSS-CA. WikiTrauma [65] is a knowledge-base website on which users collaboratively modify content and structure directly from the web, which contains free web-based knowledge tools. Wiki101 [66] is a web-based training course on how to use WikiTrauma. These two interventions were initially developed for trauma care [67], and our team’s previous work has shown that these interventions are potentially effective KT interventions to support the implementation of best practices in other fields of health care [68-70].

### Objectives

The goal of this study is to improve transition care for seniors within the CISSS-CA. Specifically, it aims to (1) implement a context-adapted CISSS-CA ACE program in its four EDs and measure the impact on patient-, caregiver-, clinical-, and hospital-level outcomes; (2) identify underlying mechanisms by which our context-adapted CISSS-CA ACE program improves care transitions for elderly people; and (3) identify underlying mechanisms by which the Wiki-suite contributes to context adaptation and local uptake of knowledge tools.

## Methods

### Study Design

**Figure 1:**
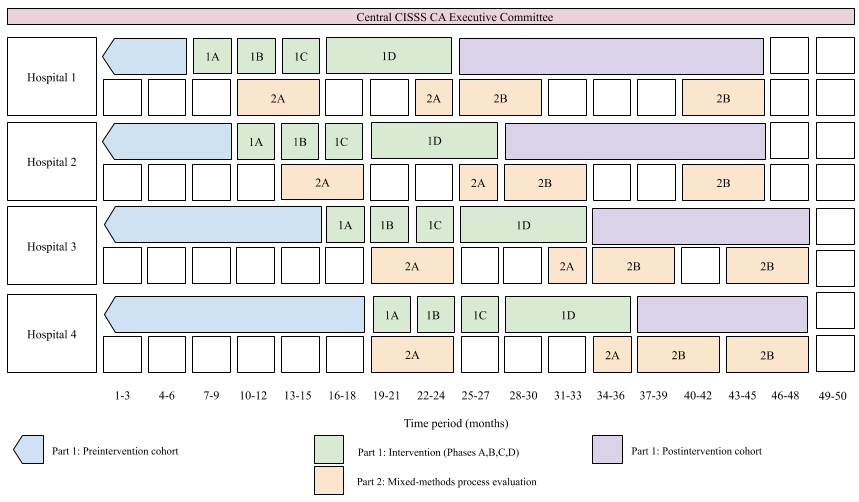
Study timeline. Staggered implementation of the context-adapted CISSS-CA ACE intervention across four hospitals and parallel mixed-methods process evaluation. ACE: Acute Care for Elders; CISSS-CA: Centre intégré de santé et de services sociaux de Chaudière-Appalaches (Chaudière-Appalaches Integrated Health and Social Services Centre).

**Figure 2:**
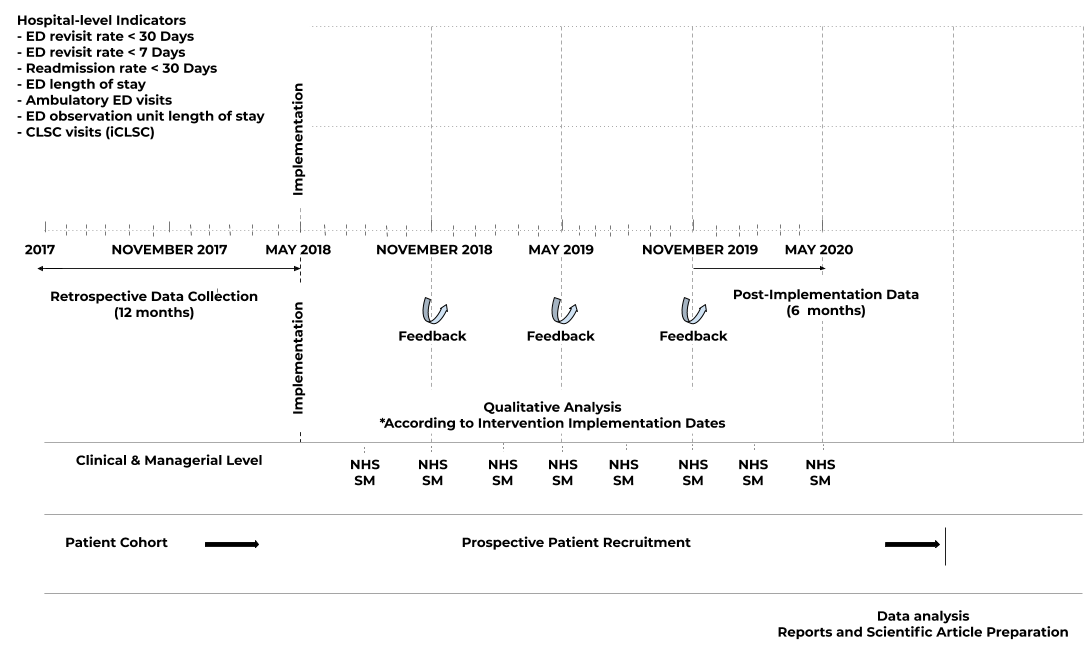
LEARNING WISDOM study design, data collection, indicators, and recruitment process. CLSC: Centres locaux de services communautaires (Local Community Services Centers); ED: emergency department; NHS SM: National Health Service Sustainability Model.

Our study will have two main parts (Figure 1). Part 1 will involve the staggered implementation of the context-adapted CISSS-CA ACE program across four CISSS-CA sites (Hôtel-Dieu de Lévis, St-Georges, Montmagny, and Thetford Mines). We will use an interrupted time series (ITS) to measure the impact of the CISSS-CA ACE program and its context-adapted tools on all hospital-level outcomes (Table 1 and Figure 2). This design will allow us to better measure the effect of our intervention on our outcomes while controlling for secular trends in our data by comparing retrospective monthly data collected in the CISSS-CA administrative databases for 36 months before our intervention and for a maximum of 12 months after our intervention in the last targeted CISSS-CA implementation site (Hospital 4 is yet to be determined) and for up to 21 months in the first targeted CISSS-CA implementation site (Hospital 1 is the Hôtel-Dieu de Lévis). Part 2 will be a parallel mixed-methods process evaluation study to understand the underlying human, organizational, and technical factors that influence the success or failure of our intervention and, more specifically, of our Wiki-suite to facilitate it through the adaptation and uptake of geriatric knowledge tools. We will report this implementation study using the Standards for Reporting Implementation Studies (StaRI) reporting guidelines (Multimedia Appendix 1) [71]. This study has been approved by the CISSS-CA Ethics Review Committee (project #2018-462, 2018-007).

**Table 1:** Primary and secondary study outcomes.

Outcomes			Data source
	
**Primary outcome**			
		Composite endpoint at each month (30-day hospital readmission and ED^a^ visit rate)	Administrative databases (Med-GPS, RAMQ^b^ billing database, and MedECHO^c^)
**Secondary outcomes**			
	**Hospital level**		
		Hospital and ED length of stay	Administrative database (Med-GPS, RAMQ billing database, and MedECHO)
		Hospital and ED admission rate	Administrative database (Med-GPS, RAMQ billing database, and MedECHO)
		Alternate level of care occupation rate	Administrative database (Med-GPS, RAMQ billing database, and MedECHO)
		Rate of patients returning to prehospital living situation	Administrative database (Med-GPS, RAMQ billing database, and MedECHO)
		Proportion of patients with family physician appointment in the 21 days after ED discharge	RAMQ billing database
	**Patient/caregiver level**		
		Quality of care transitions	Care Transition Measure (CTM-3) (48-hour postdischarge phone questionnaire)
		Functional autonomy	Chart audit to identify the PRISMA-7^d^ score and Iso-SMAF^e^ profile (case-mix classification profile according to patients’ functional autonomy characteristics as determined by the SMAF)
		Anxiety	Geriatric Anxiety Inventory-Short Form (phone questionnaire)
		Living situation at 30 days after ED discharge	Chart audit
		Burden of care	Zarit Brief Burden Interview with two additional open questions (phone questionnaire)
	**Clinical-level process**		
		Proportion of patients seen by a GEM^f^ nurse	Chart audit
		Proportion of patients/caregivers/family physicians receiving discharge summary in the 21 days following ED discharge	Postdischarge phone questionnaire and 21-day family physician follow-up phone call
		Proportion of medication patients with a reconciled medication list	Chart audit
		Proportion of eligible patients using telemonitoring services	TSS-CA^g^ telemonitoring service database

^a^ED: emergency department.

^b^RAMQ: Régie de l’assurance maladie du Québec.

^c^MedECHO: maintenance et exploitation des données pour l'étude de la clientèle hospitalière.

^d^PRISMA-7: Program of Research to Integrate the Services for the Maintenance of Autonomy.

^e^SMAF: Système de mesure de l'autonomie fonctionnelle (Functional Autonomy Measuring System).

^f^GEM: geriatric emergency management.

^g^TSS-CA: Télé-Surveillance Santé - Chaudière-Appalaches.

### Part I: Interrupted Time Series

#### Study Context

The four participating hospitals (Hôtel-Dieu de Lévis, St-Georges, Montmagny, and Thetford Mines) are part of the CISSS-CA, a new integrated health organization under the leadership of a single CEO and created in 2015 by a legislative health reform in the province of Quebec, Canada (Act to Modify the Organization and Governance of the Health and Social Services Network) [72]. The CISSS-CA has 318 health facilities within its large mixed rural and urban territory, including the four acute care hospitals in this study. Although this new supraregional organization now has the administrative infrastructure to offer integrated care, it still lacks the knowledge management infrastructure to mobilize knowledge within its organization that has 10,000 employees serving a population of more than 415,000 spread across a large territory (15,079 km^2^) [73].

#### Study Participants

##### Clinicians and Decision Makers

All hospital- and community-based clinicians and decision makers involved in care transitions will be eligible to participate. Hospital-based clinicians and decision-makers are CISSS-CA employees or independent professionals working at the participating hospitals (eg, managers, physicians, nurses, pharmacists, social workers, occupational therapists, and physiotherapists). Community-based clinicians and decision makers are CISSS-CA employees or independent professionals working outside of the hospital in a community setting (ie, home-care professionals).

##### Patients

Eligible patients will be (1) aged ≥65 years; (2) discharged from the ED; (3) able to understand and read French; and (4) able to provide informed consent.

##### Caregivers

Eligible caregivers will be identified by the patients themselves and approached only after patient consent is obtained. Caregivers will be (1) able to understand and read French and (2) able to provide informed consent.

#### Study Intervention

Our intervention is the delivery of a context-adapted CISSS-CA ACE program using a KT strategy based on our Wiki-suite. This intervention will be deployed in sequential phases.

#### Phase IA (Local Study Set-Up, 3 Months)

An executive committee will oversee the entire study (Figure 1). This committee, which will be led by the first author (PA) and Director of Nursing, will meet every month during this 4-year study. The other members include a community-based geriatric nurse specialist, a home care coordinator, the ED director, the ED head nurse, a geriatrician, a database and measurement specialist, an information technology analyst, our research coordinator, and a patient representative. A local implementation team for each participating hospital, including an ED physician, a hospitalist, a family physician, a home care nurse, an inpatient unit manager, a research assistant, and a local patient or caregiver, will also meet every month at each participating hospital starting 9 months before the active implementation of the ACE program (Phase ID). This team will include locally identified champions to lead the local implementation. Regular meetings will also be organized with local hospital and community-based clinicians and decision makers to gather relevant feedback.

#### Phase IB (Wiki101 and ACE Training, 3 Months)

All local team members and eligible health professionals or decision makers will complete the Wiki101 web-based training to learn how to navigate and edit knowledge tools in WikiTrauma. After completing Wiki101, our executive committee in collaboration with ACE experts will then offer tailored support to each local team and the local champions to implement the ACE program. The training and support aim to inform and empower the local teams by providing information on the various ACE program interventions to implement locally, the rationale for each program intervention, and the sharing of existing knowledge tools and expertise developed in each center.

#### Phase IC (Local Adaptation of Knowledge Tools, 3 Months)

The context-adapted knowledge tools created at each site will be kept in WikiTrauma [74,75]. A local working group will be created in each center. All clinicians and decision makers will be encouraged to copy, edit, and update knowledge tools within WikiTrauma to create their own context-adapted tools. Our Wiki will track all changes automatically, and we will use Google Analytics to track the use of the tools. Any changes will be reviewed during our executive committee meetings and integrated after review to ensure reliability. Partners at Mount Sinai, CFHI, and *Institut national d’excellence en santé et services sociaux* (INESSS) will also have access to these web-based tools to provide expert oversight of our tool adaptation process. For any newly developed patient-centered knowledge tools (eg, self-care management guides), we will solicit in-depth feedback from a patient representative, who will lead a subcommittee of caregiver and patient representatives from the participating sites.

#### Phase ID (Implementation, 9 Months)

In the 9 months following training and local adaptation, we will implement the context-adapted CISSS-CA ACE program with the support of WikiTrauma and local implementation teams who will have the responsibility to roll out the different elements of our intervention within their respective hospitals. Our context-adapted CISSS-CA ACE program will include a series of systematic predischarge, postdischarge, and across transition period interventions for eligible patients as follows: (1) screening of patients in need of multidimensional evaluation of their loss of autonomy (with the *Programme de Recherche sur l’Intégration des Services de Maintien de l’Autonomie 7* [PRISMA-7] tool) [76]; (2) a geriatric emergency management (GEM) nurse; (3) geriatric training [77]; (4) communication tools for transmitting information to nursing homes and other community-based stakeholders in the health system; (5) a fall prevention program; (6) systematic medication reconciliation; (7) elder-friendly ED environment adaptation; (8) access to clinician- and patient-centered Wiki-based KT tools [78,79] (eg, standing orders for geriatric patients admitted to the ED, geriatric analgesia prescription order set, and patient communication tools and decision aids); and (9) access to a community-based telemonitoring service. This service, which is offered by *Télé-Surveillance Santé - Chaudière-Appalaches* (TSS-CA) [80], will be offered for free to all eligible patients (ie, patients aged over 65 years who have at least seven ED visits in last 12 months, where over half of these visits are triaged to the ED observation unit and where two visits are not followed by hospitalization) transitioning from the hospital or ED to their home. This service currently offers remote monitoring of patients; nurses available 24 hours a day, 7 days a week, 365 days a year; and monthly phone check-ups. It also includes a customized emergency response intervention when patients are in need and connects patients with a network of community-based volunteer caregivers who are notified to visit patients when in need or simply to conduct a routine check-up.

In parallel with these interventions, we will also offer audit and feedback for health professionals and decision-makers in order to support organizational learning that will allow for real-time adjustment of interventions that are implemented. Feedback will take the form of monthly newsletters for the CISSS-CA covering patient-, clinician-, and hospital-level quality indicators. Moreover, our research team will be embedded within the CISSS-CA elder-friendly hospital committee, which plays an active role in supporting quality improvement initiatives and program implementation. This will ensure that results generated by our research team will benefit our population and ensure timely integrated knowledge translation.

#### Study Comparison

Results from the centers will be compared with their own results in the preintervention period using data collected for the ITS study.

#### Outcomes Measured

Our primary outcome will be a hospital-level composite outcome of 30-day hospital readmission and ED visit rate. Our secondary outcomes will be hospital-, clinical-, and patient- or caregiver-level outcomes (Table 1).

#### Hospital-Level Outcomes

Hospital administrative databases (eg, Med-GPS, Logibec) will be used to calculate monthly hospital-level outcomes. Monthly data will then be analyzed to form points in time. Data will be extracted from the *Régie de l’assurance maladie du Québec* (RAMQ) physician billing database and Maintenance et exploitation des données pour l'étude de la clientèle hospitalière (Med-ECHO) database (containing data on hospitalizations and health professional consultations for all institutions) in addition to databases available at the INESSS in order to identify all public health services used prior to and after the implementation of the CISSS-CA ACE program.

#### Patient- and Caregiver-Level Sociodemographics and Outcomes

Patient and caregiver baseline sociodemographic data will include age, sex, race, language, education level, family income, prehospital living situation (eg, home, intermediate nursing homes, etc), geography of residence (rural vs urban: as defined by Statistics Canada for Rural and Small Town [81,82]), and reason for hospital admission or consultation in the ED. Patient and caregiver outcomes are presented in Table 1. The living situation will be noted in the medical file when available at 30 days after discharge. Other measures that will be collected after discharge include the Care Transitions Measure-3 (CTM-3) [17,83], the Geriatric Anxiety Inventory-Short Form (GAI-SF) [84,85], and the Zarit Burden Interview (ZBI) [86]. The CTM-3 is a three-item questionnaire that measures the perceived quality of care transition on a 0-4 scale (0, fully disagree; 4, fully agree). The French version of the tool will be used [87]. The GAI-SF measures anxiety among seniors. The short version comprises five questions. The French-Canadian version of the tool has good psychometric properties [88]. The ZBI measures the burden of caregivers. The brief French version (12 questions) of the scale has good psychometric properties and is comparable to the original version [89,90].

#### Clinical-Level Process Outcomes

In order to measure the change in care processes, we will measure process outcomes such as the proportion of patients seen by the GEM nurse, proportion of patients having a medication reconciliation list, and proportion of patients receiving telemonitoring services (Table 1).

#### Patient and Caregiver Recruitment

After receiving ethics approval, we will start recruitment for our patient-level outcomes at least 3 months before the implementation of phase 1A at each site. Due to the pragmatic nature of our study, some sites will have longer periods of patient-level outcomes collected before phase 1A than others. Patient recruitment will continue throughout our study and up to a maximum of 24 months after the intervention. We will recruit consecutive eligible ED patients aged 65 years or older, as well as one caregiver whenever possible. We will recruit 38 patients or caregivers per month for each of the four hospitals based on a precision estimate of the CTM-3 measured for the study point of each month.

With authorization from the Director of Nursing and the Professional Services Director, a member of our embedded research team will contact by telephone a randomly selected daily sample of patients who visited the ED in the last 24 hours and up to a maximum of 7 days after their ED visit. This research assistant will administer the CTM-3 questionnaire to patients for quality improvement purposes. The research assistant will then ask the patients if they agree to be contacted a second time by a member of the research team to answer additional questions for the purpose of our study. Consenting patients will then be contacted within 7 days to obtain their verbal consent to participate in the study and consent to access their medical charts. We will ensure patients’ understanding by asking them to summarize in their own words the objectives of the study and what their participation involves using the Nova Scotia criteria [91]. The team member will then collect baseline sociodemographic data and administer the questionnaires for the study. The research team will then send a written consent form by mail after the interview. If a patient refuses to be contacted by our research team or to participate in this study, the CTM-3 data collected will only be available to the CISSS-CA. The research assistant will also ask permission to contact a caregiver. Identified caregivers will be contacted by telephone to complete the Zarit questionnaire. Our research assistant will first obtain the caregivers’ verbal consent to participate in the study and then ensure the caregivers’ understanding by asking them to summarize in their own words the objectives of the study and what their participation implies.

#### Sample Size and Statistical Analysis

We will use segmented regression statistics to measure the changes in the level and slope in the postintervention period compared with the preintervention period in each center for each of our primary and secondary outcomes [92]. Thus, we will present a regression model with different intercept and slope coefficients for the pre- and postintervention time periods for each center. We will compare the changes in a composite primary outcome (total 30-day hospital readmission and ED visit rate) at our four intervention centers. We will use a Durbin-Watson test to verify the presence of autocorrelation and use an autoregressive error model to correct for this serial correlation. For an ITS, 10 measurement points before and 10 points after an intervention provide 80% power to detect a change in the level of 5 standard deviations (of the predata) only if the autocorrelation is greater than 0.4 (ie, extent to which data collected close together in time are correlated with each other) [93].

We have calculated the sample size for our patient cohort, which will be based on the smallest clinically significant difference (ie, 11%) that the CTM-3 can capture on a maximum score of 100. To detect a difference of 11% with a type I error of 5% and a type II error of 20%, 38 patients per month are needed to calculate a monthly CTM-3 estimate, based on a previous study in which CTM-3 was measured in 21 patients at Hôtel-Dieu Hospital in Lévis and showed an average of 75% and a standard deviation of 23%. Considering a call rejection rate of 70%, the number of people to be contacted in total will be 127 patients per month for each ED. We will therefore contact 5 patients per day in each of the four participating centers to obtain our targeted sample size of 38 patients at the end of the month. To ensure the creation of a random and representative sample of our population of elderly patients being discharged from the ED, a randomization table based on all patients discharged in the previous 24 hours from the ED will be provided every day to select which patients to call.

### Part II: Parallel Mixed-Methods Process Evaluation

Part II aims to identify the underlying mechanisms (human, organizational, and technical) by which our context-adapted CISSS-CA ACE intervention improves care transitions for elderly people and, more specifically, how the Wiki-suite contributes to context-adaptation and local uptake of knowledge tools. Contextual elements affecting the implementation of the ACE program at the four hospital sites and use of our Wiki-suite cannot be addressed using ITS methodology, yet they can have a relevant impact on the use [94]. To scale up the CISSS-CA ACE program and Wiki-suite in multiple settings, we need to understand these contextual elements.

#### Approach

##### Phase IIA (Measurement of the Intention to Use WikiTrauma and Actual WikiTrauma Use, 9 Months)

Alongside phases IB and IC, we will conduct a theory-based process evaluation. All Wiki101 participants will be invited to answer a validated Theory of Planned Behavior questionnaire [95] at baseline before Wiki101, immediately after Wiki101, and at the end of Phase ID to measure the change in intention and the impact of Wiki101 on Theory of Planned Behavior determinants. We will also use Google Analytics to track the use of WikiTrauma tools by the participants over time. Segmented regression statistics will be used to measure the changes in the level and slope for postintervention Wiki use compared with preintervention Wiki use [92].

##### Phase IIB (Stakeholder Interviews, 6 Months)

We will conduct at least 32 45-minute individual interviews with purposefully selected key informants (a minimum of two clinicians, two managers, and four patients and/or caregivers per center) to identify the contextual elements influencing the successful (or failed) implementation of the ACE program for improving care transitions and understand how our Wiki-suite facilitated this. These interviews will take place at least 9 months after first implementing the ACE program at each hospital. A pool of eligible health professionals working in the ED and administrators or decision makers will be created by direct solicitation during departmental or staff meetings (upon invitation from department chiefs) or by direct solicitation by department chiefs. Our study’s goals and procedures will be briefly explained, including eventual solicitation to participate in individual interviews. All eligible and consenting individuals will be asked to provide their contact information for eventual solicitation. Participants will be identified from the pool described above, using purposeful sampling, with an emphasis on maximum variation to obtain a wide range of different points of view and opinions. Each selected individual will be solicited via the contact information provided at initial solicitation. A consent form will be completed over the internet prior to the phone interview. These interviews will be confidential, and no information allowing the identification of individuals will be provided to the administration of the CISSS-CA.

A PhD student guided by experienced qualitative researchers will perform the interviews and process evaluation of our intervention. For this analysis, we will perform a mixed inductive and deductive qualitative content analysis of the verbatim transcripts and field notes taken during these interviews, as well as key implementation project documents such as executive committee and local team meeting minutes [94,96]. The analysis will involve reading the verbatim transcripts and key project documents thoroughly and developing codes that represent the nature of the implementation, adaptation processes at each site, and barriers and facilitators to using our context-adaptation methodology. Our previous experience in conducting qualitative content analysis about Wiki use will help us understand how our Wiki-based intervention succeeded (or not) in improving care transitions [70]. We will use the Ottawa Model for Research Use [97] and the KTA framework [98] to structure our analysis.

We will also use the National Health Service (NHS) Sustainability Model to guide our process evaluation analysis [99]. The NHS Sustainability Questionnaire has been developed to support health care leaders to implement and sustain effective improvement initiatives in health care systems. The questionnaire is a diagnostic tool that identifies strengths and weaknesses in the implementation plan and predicts the likelihood of sustainability for improvement initiatives. The NHS Sustainability Questionnaire will be administered to members of the executive committee and each local implementation team at baseline before phase IA and at regular 3-month periods until 12 months after the end of phase ID.

##### Phase IIC (Comparative Analysis of Case Studies, 6 Months)

We will analyze the impact of our intervention within the context of Quebec’s health reform aiming at better integration of care within the health system [100]. This will be accomplished by conducting a comparative case study across the four study sites to compare the barriers, facilitators, and local solutions implemented to gain a better understanding about how our ACE program and Wiki-suite–mediated intervention could eventually be scaled up elsewhere.

## Results

This study was funded by the Canadian Institutes for Health Research in May 2017. The project was approved by the CISSS-CA ethics committee in May 2018. Data collection started in January 2019. As of January 2020, we enrolled 1635 patients and 529 caregivers from the four participating hospitals. Data collection is projected to be completed in January 2022. Data analysis has not yet begun. Results are expected to be published in 2022. Expected results will be presented to different key internal stakeholders to better support efforts and resources deployed in the transitions of seniors. Through key interventions focused on seniors, we are expecting to increase patient satisfaction and quality of care and reduce readmission and ED revisit.

## Discussion

### Principal Findings

Our study will produce a new partnership among patients, clinicians, and decision makers, engaging and empowering them to improve care for elderly people by implementing the CISSS-CA ACE program in four hospitals within the CISSS-CA. This study will also provide qualitative and quantitative evidence on effective strategies for improved transition care for elderly people. This study specifically aims at decreasing CISSS-CA’s 30-day rate of readmission for elderly patients, which has been increasing since 2014 (13% in 2014 and 16% in 2016), and its high 30-day ED visit rate (21% in 2014 and 22% in 2016). Our team will generate real-time quality improvement data that will guide decision-making by the CISSS-CA and help adjust resource allocation and policy-making that will positively influence future care transitions for elderly people.

Our study will also identify the human, organizational, and technical factors that support the local adaptation of knowledge and the scale-up of our intervention in other care settings within the CISSS-CA and elsewhere in Canada [101,102]. This will contribute to making the CISSS-CA a learning organization and to training a new cadre of clinicians, administrators, policy-makers, and scientists who will transform our health system [103]. This is also highly relevant to INESSS and CFHI, who aim to scale up innovations and best practices in the care of seniors in Quebec and Canada, respectively.

### Limitations

Our study is measuring the impact of a complex intervention, which is a common problem in health services research. The ability to measure and evaluate the effect of complex interventions remains underdeveloped [104-106]. The Medical Research Council suggests evaluating complex interventions using experimental designs when possible, but quasi-experimental designs, such as prospective pre/post cohort studies [107,108] and interrupted time series [93,109,110], are acceptable when randomization is not possible, feasible, or ethical [111,112]. The highly iterative nature of our intervention made a cluster stepped wedge trial and other experimental designs unfeasible. An advantage of using an interrupted time series design is that it allows for the statistical investigation of potential biases in the estimate of the effect of the intervention. These potential biases include secular trends, seasonal variations, duration of the intervention, random fluctuations, and autocorrelation [93]. Thus, for feasibility reasons, we chose to apply an interrupted time series design for our primary outcome because we could easily access data for hospital-level outcomes using administrative databases. This interrupted time series design will be applied only if our descriptive statistics allow us to detect a clear inflection point that correlates with the implementation of our complex intervention. Our mixed-methods process evaluation will also help us understand the mechanisms in play during our study and detect any negative experiences.

Another challenge to complex interventions is the lack of local adoption. In the design of our intervention, we have incorporated best practices from the field of implementation science to design a standardized intervention comprising local barrier identification [113], multidisciplinary teamwork [114], local adaptation [115], and use of local champions [116]. We chose the ACE program because it has a strong theoretical background supporting its use and because systematic reviews support the effectiveness of its interventions [42-48]. Our strong decision- and policy-maker buy-in, frequent data collection and tracking, iterative and collaborative design, and frequent consultation with local stakeholders including patients will allow our team to identify challenges and mitigation strategies by discussing plans with team members and external partners or mentors at CFHI, INESSS, and Mount Sinai Hospital. Our budget has stipends to support the planned research activities.

### Conclusion

This study will provide much needed evidence on effective KT strategies to adapt best practices to the local context in the transition of care for elderly people. It will contribute to adapting geriatric knowledge to the local context. The knowledge generated through this study will support future scale-up of ACE programs and our Wiki methodology in other settings in Canada.

## Appendix

Multimedia Appendix 1 Standards for reporting implementation studies (StaRI) checklist.

## Abbreviations

ACE: Acute Care for Elders
CEO: Chief Executive Officer
CFHI: Canadian Foundation for Healthcare Improvement
CISSS-CA: Centre intégré de santé et de services sociaux de Chaudière-Appalaches (Chaudière-Appalaches Integrated Health and Social Services Centre)
CTM-3: Three-item Care Transitions Measure
ED: emergency department
GAI-SF: Geriatric Anxiety Inventory-Short Form
GEM: geriatric emergency management
INESSS: Institut national d'excellence en santé et services sociaux
ITS: interrupted time series
KT: knowledge translation
KTA: knowledge-to-action
NHS: National Health Service
PRISMA: Programme de recherche sur l’intégration des services de maintien de l’autonomie (Program of Research to Integrate the Services for the Maintenance of Autonomy)
RAMQ: Régie de l'assurance maladie du Québec
TSS-CA: Télé-surveillance santé-Chaudière-Appalaches (Health telemonitoring and structured telephone support in Chaudière-Appalaches)
ZBI: Zarit Burden Interview
